# Twist1-induced epithelial-mesenchymal transition according to microsatellite instability status in colon cancer cells

**DOI:** 10.18632/oncotarget.10974

**Published:** 2016-08-01

**Authors:** Bo Young Oh, So-Young Kim, Yeo Song Lee, Hye Kyung Hong, Tae Won Kim, Seok Hyung Kim, Woo Yong Lee, Yong Beom Cho

**Affiliations:** ^1^ Department of Surgery, Samsung Medical Center, Sungkyunkwan University School of Medicine, Seoul, Korea; ^2^ Samsung Biomedical Research Institute, Samsung Medical Center, Seoul, Korea; ^3^ Department of Pathology, Samsung Medical Center, Sungkyunkwan University School of Medicine, Seoul, Korea; ^4^ Department of Health Sciences and Technology, SAIHST, Sungkyunkwan University, Seoul, Korea; ^5^ Department of Medical Device Management & Research, SAIHST, Sungkyunkwan University, Seoul, Korea

**Keywords:** Twist1, epithelial to mesenchymal transition, microsatellite instability, colorectal cancer

## Abstract

Colorectal cancer (CRC) with microsatellite instability (MSI) may exhibit impaired epithelial-mesenchymal transition (EMT), but little is known about the underlying mechanisms of this phenomenon. In this study, we investigated the role of Twist1 and its downstream signaling cascades in EMT induction according to MSI status. To investigate the effects of Twist1 on EMT induction according to MSI status, MSS LS513 and MSI LoVo colon cancer cell lines, which overexpress human Twist1, were generated. Twist1-induced EMT and its downstream signaling pathways were evaluated via *in vitro* and *in vivo* experiments. We found that Twist1 induced EMT markers and stem cell-like characteristics via AKT signaling pathways. Twist1 induced activation of AKT and suppression of glycogen synthase kinase (GSK)-3β, which resulted in the activation of β-catenin, increasing CD44 expression. In addition, Twist1 activated the AKT-induced NF-κB pathway, increasing CD44 and CD166 expression. Activation of both the AKT/GSK-3β/β-catenin and AKT/NF-κB pathways occurred in MSS LS513 cells, while only the AKT/GSK-3β/β-catenin pathway was activated in MSI LoVo cells. In conclusion, Twist1 induces stem cell-like characteristics in colon cancer cell lines related to EMT via AKT signaling pathways, and those pathways depend on MSI status.

## INTRODUCTION

Epithelial-mesenchymal transition (EMT) is a highly conserved process characterized by the loss of epithelial characteristics and the acquisition of mesenchymal phenotypes [1]. In cancer, tumor cells undergoing EMT gain motility, invasiveness, and stem cell-like characteristics, resulting in cancer progression and metastasis [2–4]. During EMT, tumor cells undergo a loss of epithelial markers, such as E-cadherin, and a gain of mesenchymal markers, such as vimentin and N-cadherin [1, 4, 5]. EMT is induced by transcription factors such as Twist, snail, ZEB, and FOXC [1, 5]. In particular, Twist1 is a key regulator of EMT and a potent promoter of cancer progression and metastasis [6–8], although the underlying mechanisms are poorly understood.

Metastasis is the most common cause of death in colorectal cancer (CRC) patients, which occurs in approximately 50% of patients during the course of the disease [9–11]. Therefore, many studies have sought to understand the molecular mechanisms of metastasis and have proposed EMT as a candidate mechanism of metastasis in CRC [12–15]. A recent study suggested that EMT is impaired in colon cancer cells with microsatellite instability (MSI) [4]. MSI results from defective DNA mismatch repair, one of the major mechanisms of carcinogenesis [16, 17]. The MSI phenotype is associated with approximately 10-15% of sporadic CRC and almost all hereditary non-polyposis CRC (HNPCC) [16–18]. Many studies have reported that CRCs with MSI show less frequent metastasis and better prognosis compared to microsatellite stable (MSS) CRC [18, 19]. However, little is known about the molecular pathogenesis of prognostic differences between MSI and MSS CRC. Thus, investigating the morphological and functional differences of Twist1-induced EMT pathways in MSS and MSI CRC may help elucidate the molecular mechanisms of metastasis in CRC. In this study, we investigated the role of Twist1 and its downstream signaling cascades for inducing EMT in MSS and MSI colon cancer cell lines.

## RESULTS

### Induction of EMT by Twist1 in MSS and MSI colon cancer cell lines

**Figure 1:**
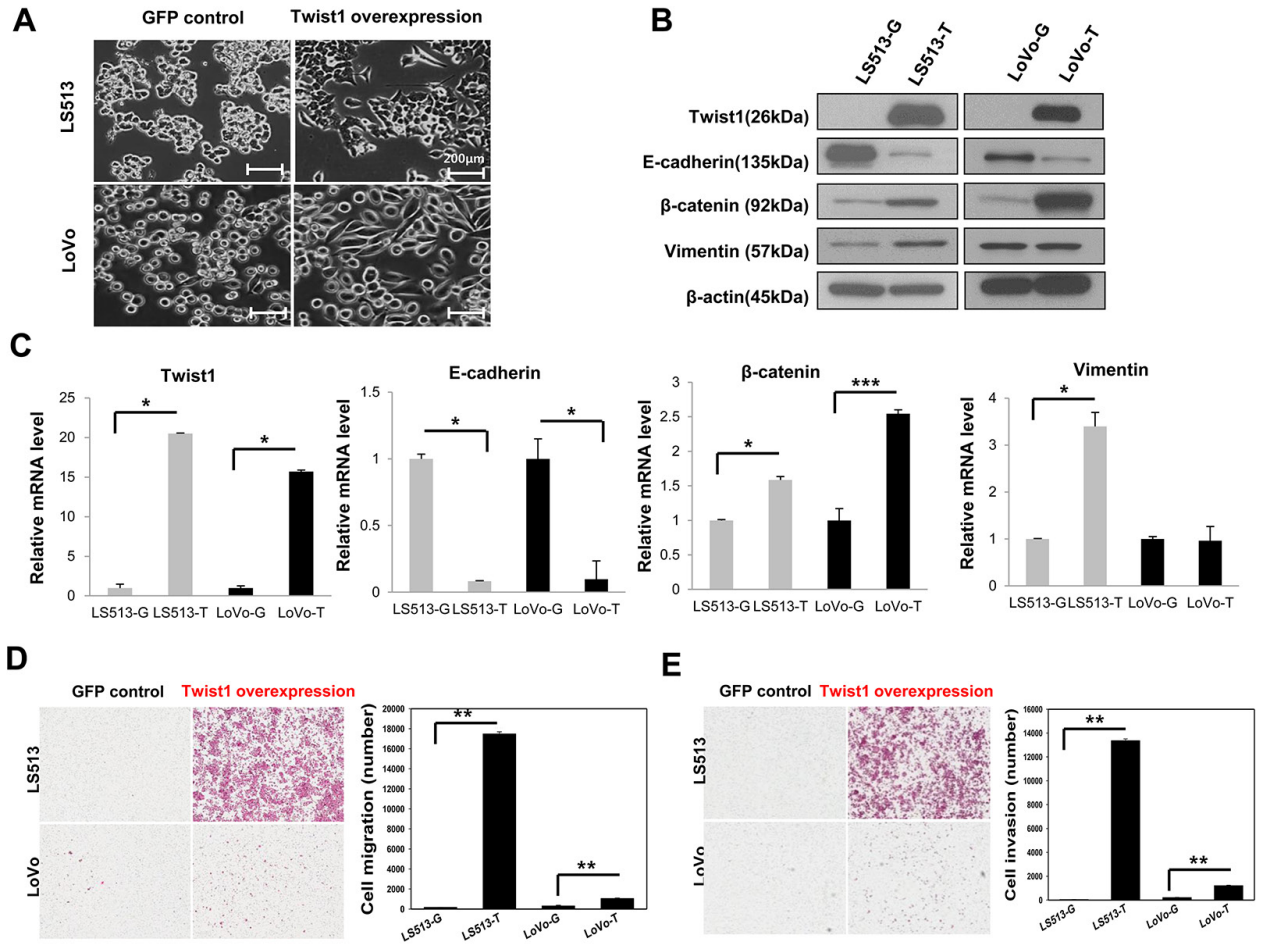
Twist1 induced EMT in MSS LS513 and MSI LoVo colon cancer cell lines EMT induction was more prominent in MSS LS513 compared to MSI LoVo cells. **A.** Cellular morphologic changes depending on Twist1 overexpression are shown at 40x magnification. Scale bar: 200 μm. **B.** Expression of E-cadherin, β-catenin, and vimentin depending on Twist1 overexpression was analyzed by western blot. β-actin was used as a loading control. **C.** Transcriptional levels of E-cadherin, β-catenin, and vimentin depending on Twist1 overexpression were analyzed by real-time PCR. **D.** Cell migration depending on Twist1 overexpression was assessed by transwell migration chamber assay. **E.** Cell invasiveness depending on Twist1 overexpression was assessed by transwell migration chamber assay. Columns = mean ± SD (n = 3). * p < 0.05, ** p < 0.01, *** p < 0.001.

To investigate the effect of Twist1 on EMT induction according to MSI status, MSS LS513 and MSI LoVo cells overexpressing human Twist1 were generated via lentivirus transduction. EMT induction appeared in these cells with morphological changes such as loss of cell polarity, spindle-like cell shape, and loss of cell-to-cell adhesion. In contrast, epithelial features still remained in control green fluorescent protein (GFP)-expressing cells (Figure 1A). We carried out western blotting to identify EMT markers depending on Twist1 overexpression. Twist1 overexpression induced loss of the epithelial marker E-cadherin, which is involved in the early stages of EMT, and then loss of the E-cadherin promoted β-catenin release in both MSS LS513 and MSI LoVo cells. However, upregulation of mesenchymal marker vimentin was prominent only in MSS LS513 cells (Figure 1B). Similar results were noted by real-time PCR (Figure 1C).

In addition, to investigate the effect of Twist1 on cancer progression according to MSI status, we evaluated cell migration and cell invasion in MSS LS513 and MSI LoVo cells. Twist1-overexpressing cells had more mobile and invasive properties than control GFP-expressing cells, and these functional changes were more prominent in MSS LS513 than in MSI LoVo cells (Figures 1D and 1E).

### Activation of AKT signaling pathway by Twist1 in MSS and MSI colon cancer cell lines

**Figure 2:**
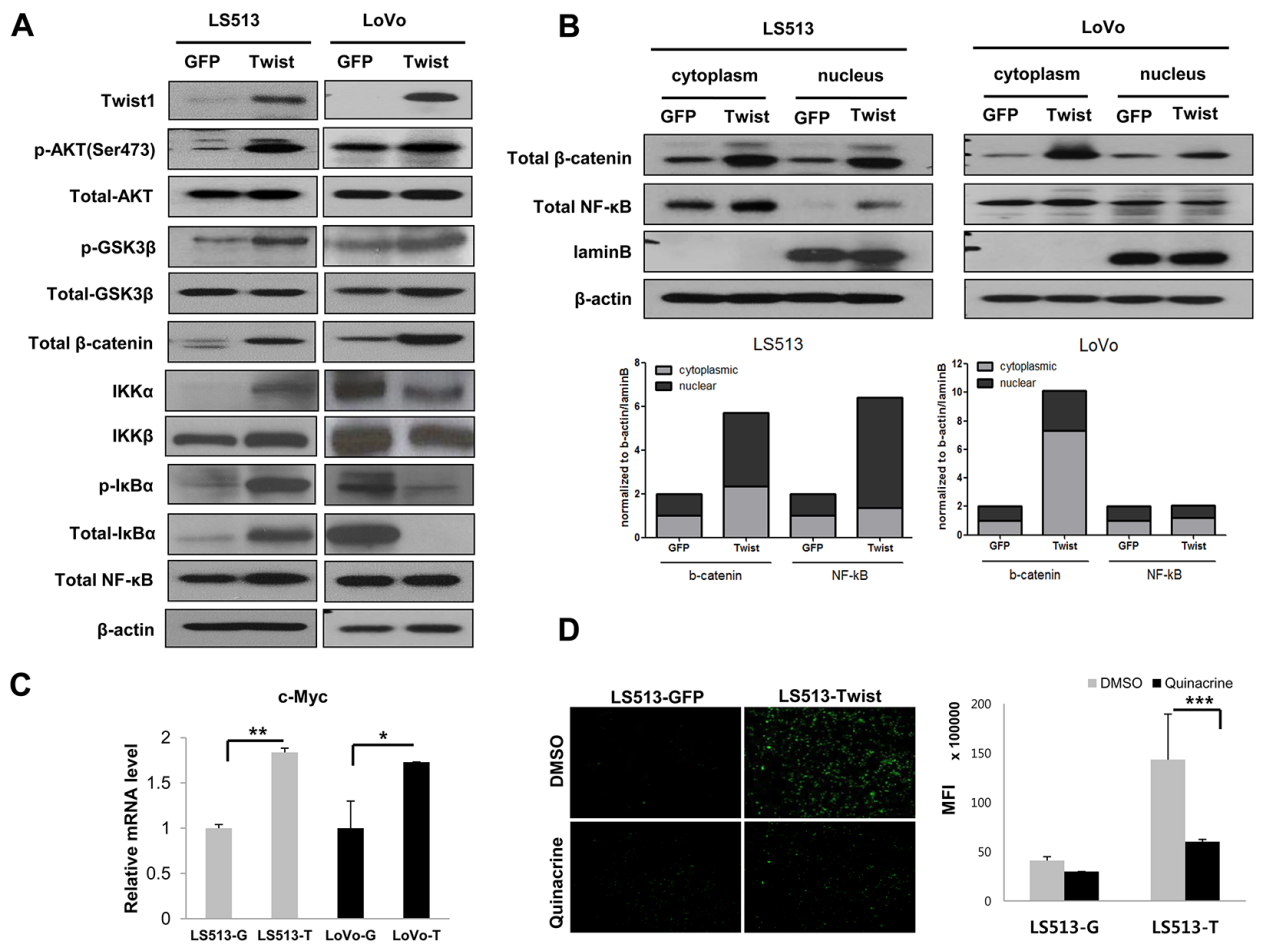
Twist1 activated the AKT signaling pathway in MSS LS513 and MSI LoVo colon cancer cell lines The AKT/GSK-3β/β-catenin pathway was activated in MSS LS513 and MSI LoVo cells, and the AKT/NF-κB pathway was activated in MSS LS513 cells. **A.** Expression of molecules involved in the AKT/GSK-3β/β-catenin pathway and AKT/NF-κB pathway depending on Twist1 overexpression was analyzed by Western blot. **B.** Subcellular localization of β-catenin and NF-κB depending on Twist1 overexpression was analyzed in each cell line. **C.** Transcriptional levels of c-Myc, a target of β-catenin, depending on Twist1 overexpression were analyzed via real-time PCR. **D.** Inhibition of NF-κB by quinacrine suppressed invasiveness of Twist1-overexpressing MSS LS513 cells. Representative images of invading cells are shown (upper panel), and the mean fluorescence intensities (MFIs) of invaded areas under the various conditions are presented in bar graphs (lower panel). Columns = mean ± SD (n = 3). * p < 0.05, ** p < 0.01, *** p < 0.001.

Twist1 has been shown to activate AKT expression and its downstream signaling pathway [20]. To investigate the effect of Twist1 on activation of the AKT downstream signaling pathway according to the MSI status, we evaluated the phosphorylation of AKT (Ser473) in MSS LS513 and MSI LoVo cells. In addition, expression of the glycogen synthase kinase (GSK)-3β (Tyr216) inactive form, which is a downstream target of AKT, and β-catenin were evaluated. We found that Twist1 induced activation of AKT and suppression of GSK-3β (which resulted in nuclear translocation of β-catenin) in MSS LS513 cells (Figures 2A and 2C). In addition, in MSS LS513 cells overexpressing Twist1, AKT activated inhibitor of κB kinase (IKK), which activated the nuclear factor kappa-light-chain-enhancer of activated B cells (NF-κB) via phosphorylation of total inhibitor of κB (IκB) binding on NF-κB, thereby allowing active NF-κB to translocate into the nucleus (Figures 2B and 2C). However, similar findings were not observed in MSI LoVo cells. In LoVo cells, Twist1 activated AKT and suppressed GSK-3β, which then led to nuclear translocation of β-catenin; however, it did not activate NF-κB via the phosphorylation of IκB (Figures 2A-2C).

Furthermore, we investigated whether inhibition of NF-κB signaling suppressed invasive properties of Twist1-overexpressing cells. NF-κB-activating LS513 cells were treated with 2 μM quinacrine, an NF-κB inhibitor, for 24 hours, and cell invasion assay was then performed. Quinacrine treatment led to a significant decrease in cell invasiveness in Twist1-overexpressing LS513 cells (Figure 2D). These findings suggest that Twist1 facilitates AKT-induced NF-κB pathway, increasing cancer cell invasiveness.

### Induction of stem cell-like characteristics by Twist1 in MSS and MSI colon cancer cell lines

**Figure 3:**
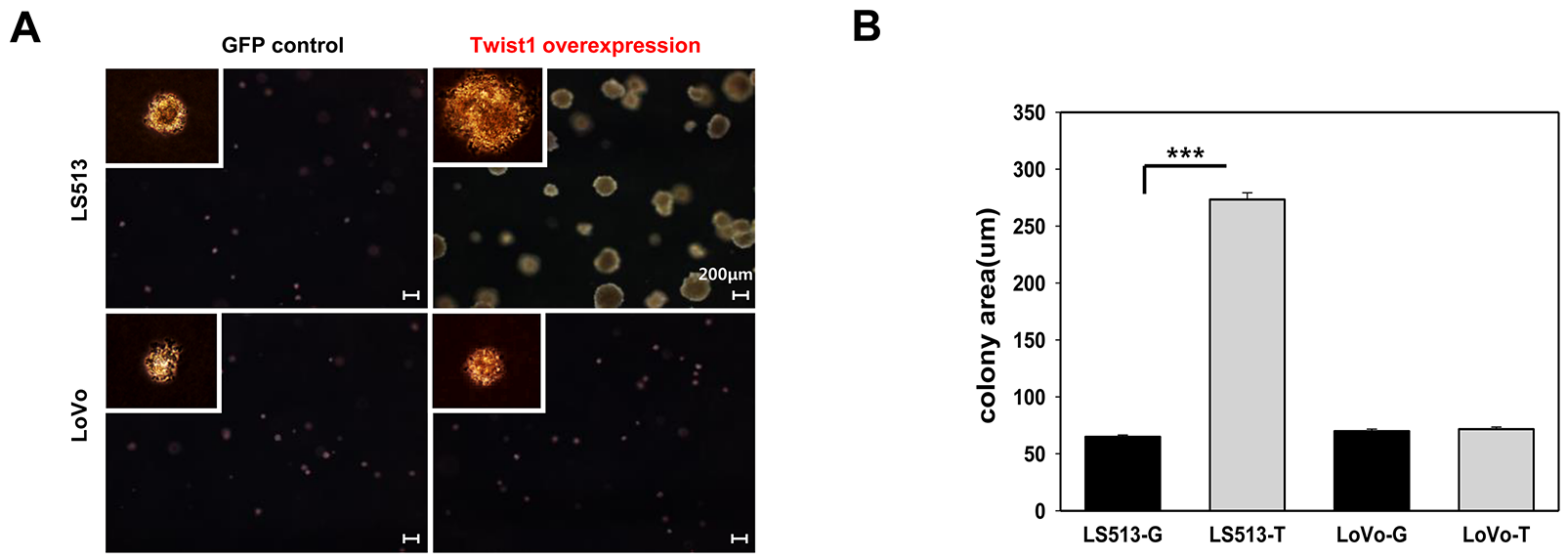
Twist1 enhanced colony formation in MSS LS513 cells compared to in MSS LoVo cells **A.** The ability of cells to self-renew was assessed via colony formation. The white rectangle in the spherical formation image denotes a region shown at 20x magnification. Scale bar: 200 μm. **B.** Cell growth was quantified based on the number and size of colonies. Columns = mean ± SD (n = 3). * p < 0.05, ** p < 0.01, *** p < 0.001.

To examine Twist1-induced stem cell-like characteristics, we investigated the ability of cells to self-renew by evaluating colony formation. Twist1 enhanced colony formation in MSS LS513 cells, but this finding was not marked in MSI LoVo cells (Figure 3).

**Figure 4:**
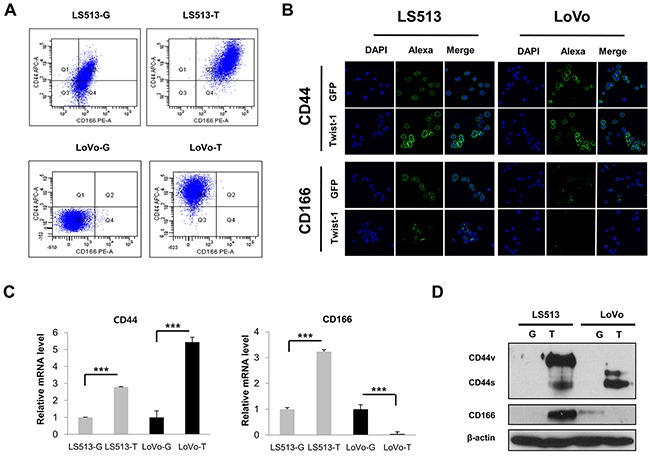
Twist1 elevated the level of cancer stem cell markers in MSS LS513 and MSI LoVo colon cancer cell lines Expression of CD44 was higher in MSS LS513 and MSI LoVo cells, and expression of CD166 was higher in MSS LS513 cells. **A.** Expression of CD44 and CD166 depending on Twist1 overexpression was analyzed by FACS. **B.** Expression of CD44 and CD166 depending on Twist1 overexpression was analyzed by immunofluorescent staining; original magnification, 400x. **C.** Transcriptional levels of CD44 and CD166 depending on Twist1 overexpression were analyzed by real-time PCR. **D.** Expression of CD44 variant isoforms (CD44v), CD44 standard isoforms (CD44s), and CD166 depending on Twist1 overexpression was analyzed via immunoblot. Columns = mean ± SD (n = 3). * p < 0.05, ** p < 0.01, *** p < 0.001.

To further confirm these findings, we also evaluated the expression of stem cell markers, such as CD44 and CD166, by using fluorescence-activated cell sorting (FACS), immunofluorescence staining, real-time PCR, and immunoblotting. As shown in FACS, Twist1 elevated the level of CD44 and CD166 expression in MSS LS513 cells. However, in MSI LoVo cells, Twist1 promoted the expression of CD44, but not CD166 (Figure 4A). Immunofluorescence staining, real-time PCR, and immunoblotting confirmed that expression of both CD44 and CD166 was increased in Twist1-overexpressing MSS LS513 cells, but only CD44 expression was increased in Twist-overexpressing MSI LoVo cells (Figures 4B-4D).

**Figure 5:**
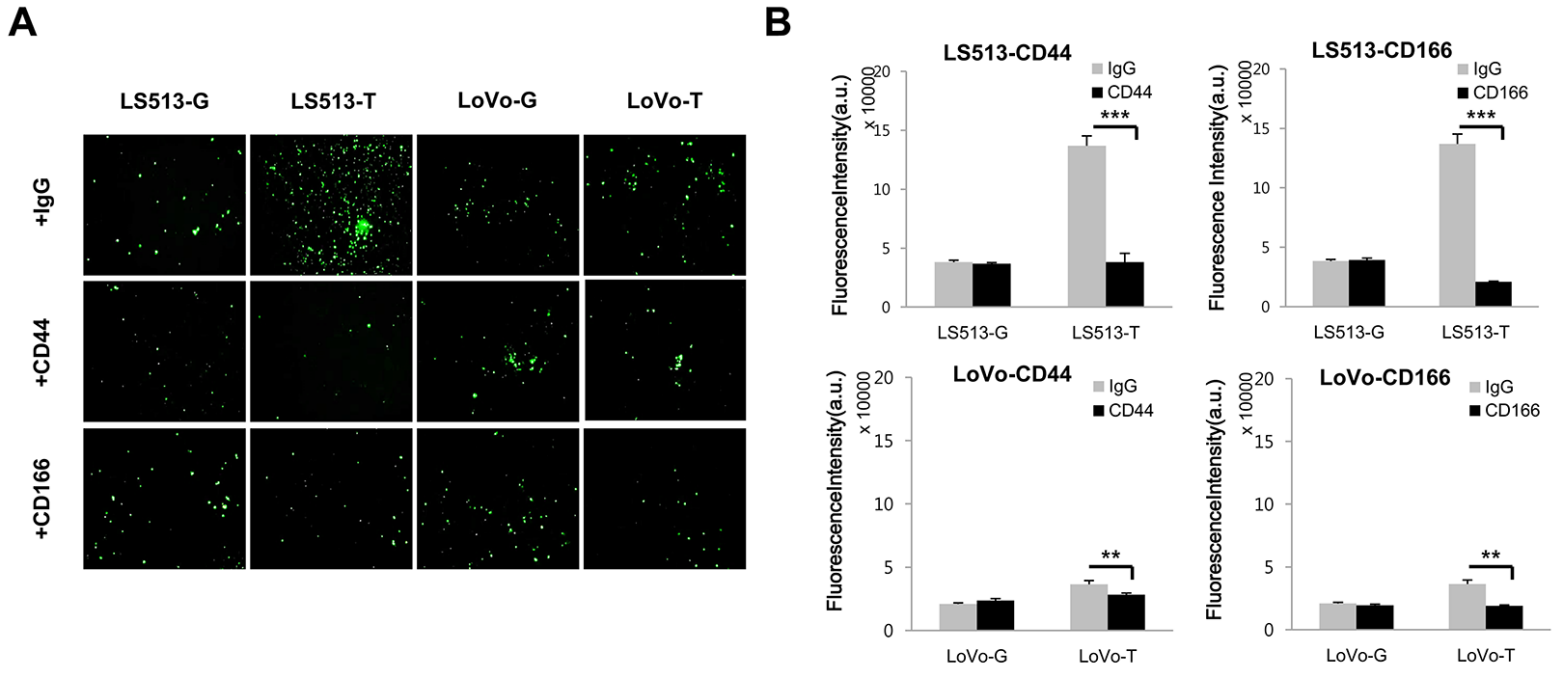
Invasion assay was performed by adding neutralizing antibodies against CD44 and CD166 in MSS LS513 and MSI LoVo colon cancer cell lines The inhibiting effects of neutralizing antibodies were marked in MSS LS513 cells, but were minimal in MSI LoVo cells. **A.** Representative images of invading cells are shown. **B.** The mean fluorescence intensities (MFIs) of invaded areas under the various conditions are presented. Columns = mean ± SD (n = 3). * p < 0.05, ** p < 0.01, *** p < 0.001.

In addition, we investigated whether expression of CD44 and CD166 was associated with cancer cell invasiveness. Neutralizing antibodies against human CD44 and CD166 were added to Twist1-overexpressing cells and a cell invasion assay was then performed. Neutralizing antibodies against CD44 and CD166 inhibited the invasiveness of cancer cells in MSS LS513 cells. However, these inhibiting effects were not marked in MSI LoVo cells (Figure 5).

### Twist1-induced tumorigenesis in xenografts with MSS and MSI colon cancer

**Figure 6:**
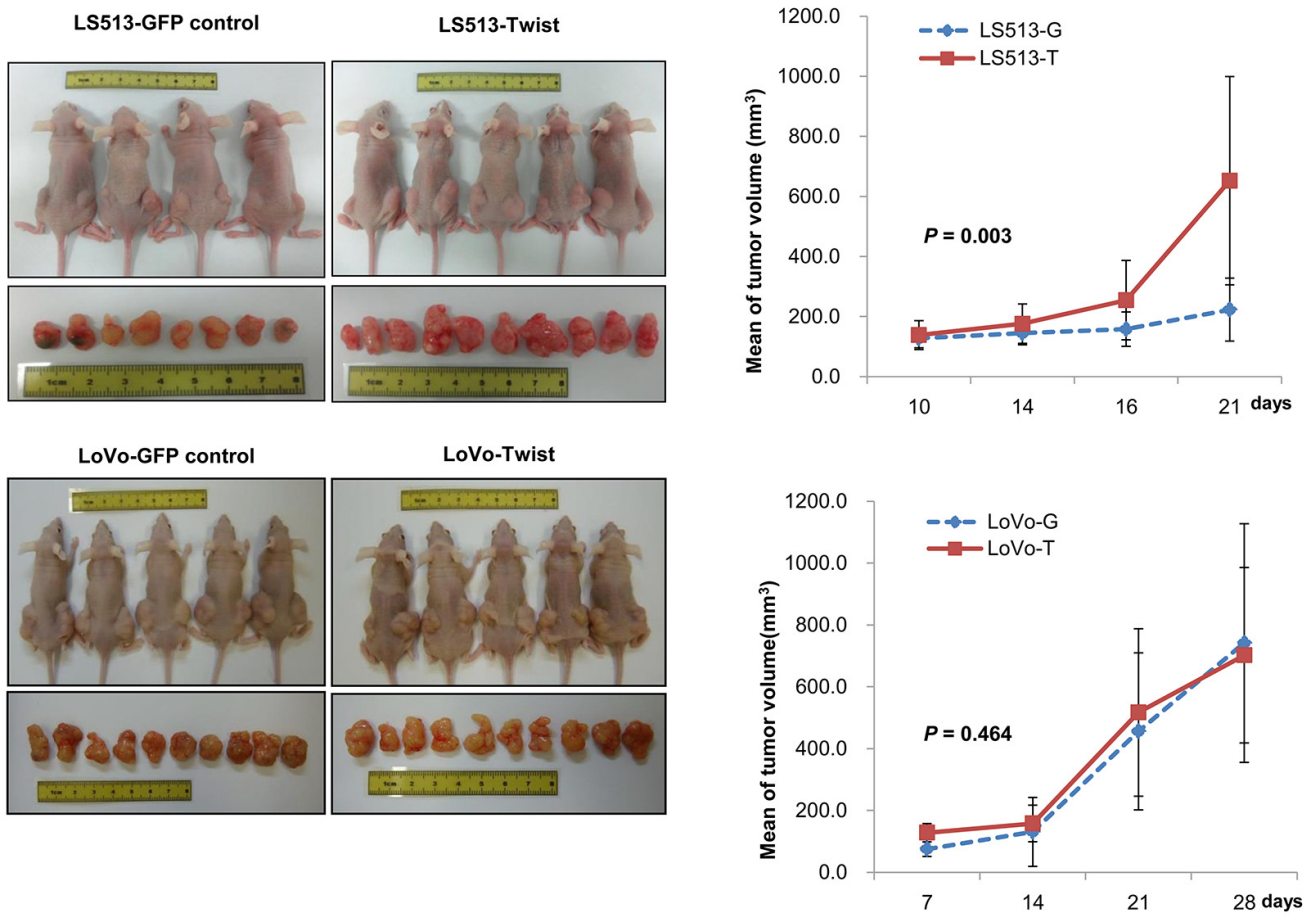
Twist1-induced tumorigenesis in xenografts varied according to MSI status Tumorigenesis was more prominent in Twist1-overexpressing MSS LS513 tumors compared to GFP-expressing tumors. In contrast, tumorigenesis was not increased in Twist1-overexpressing LoVo cells compared to GFP-expressing LoVo cells. Injected cell counts = 5 × 10^6^/100 μl.

To investigate Twist1-induced tumorigenesis *in vivo* according to MSI status, we generated xenografts by implanting MSS LS513 and MSI LoVo colon cancer cells. Consistent with *in vitro* findings, Twist1-overexpressing MSS LS513 tumors displayed more prominent tumorigenesis compared to GFP-expressing tumors. Between one and three weeks, mean tumor volume increased from 100 to 650 mm^3^ in Twist1-overexpressing LS513 tumors and from 100 to 200 mm^3^ in control LS513 tumors (*P* = 0.003). Tumors grew rapidly after the initial period passed, suggesting that Twist1 affects the late period of tumor development in MSS LS513 tumors (Figure 6). In contrast, as shown in Figure 6, Twist1 did not induce tumorigenesis in MSI LoVo tumors. This outcome was consistent with *in vitro* findings that activation of the Twist1-induced AKT/NF-κB pathway promoting invasion and tumorigenesis was not observed in MSI LoVo cells.

## DISCUSSION

**Figure 7:**
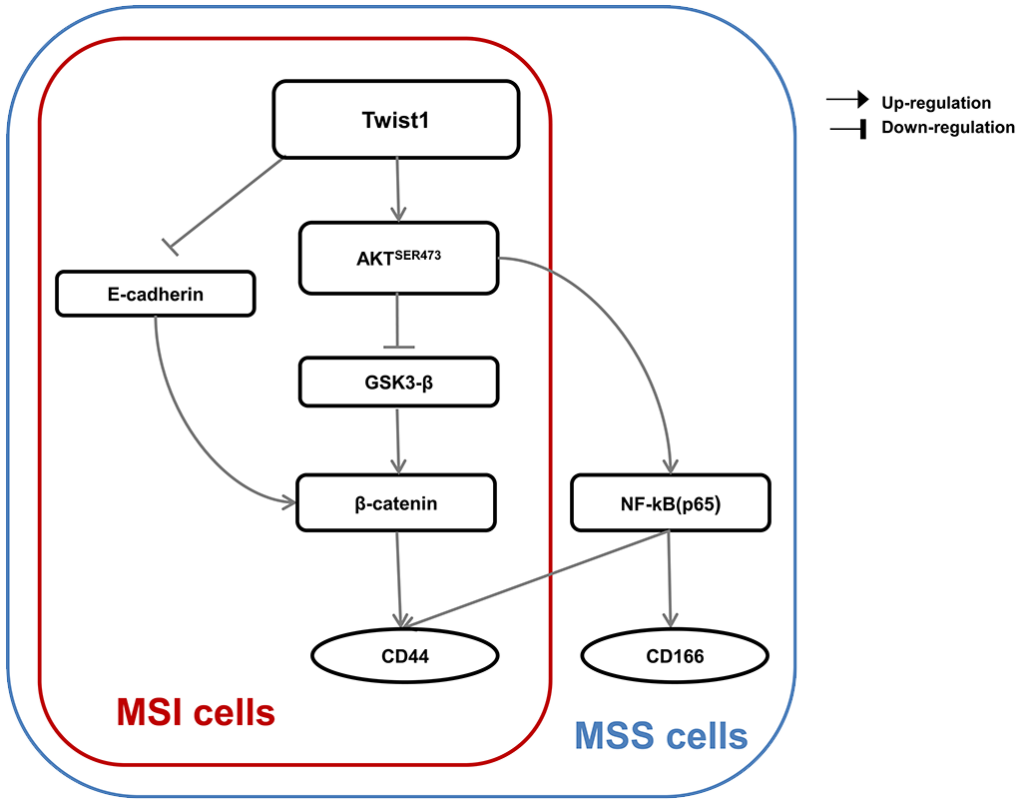
A proposed model for a Twist1-induced EMT signaling pathway according to MSI status is presented In MSS LS513 cells, the AKT/GSK-3β/β-catenin pathway and AKT/NF-κB pathway activated. In contrast, in MSI LoVo cells, only the AKT/GSK-3β/β-catenin pathway activated.

In this study, we examined whether Twist1 induced stem cell-like characteristics by EMT via AKT signaling pathways in colon cancer cell lines and if those pathways depended on MSI status. First, Twist1 activated AKT-induced NF-κB pathway, increasing CD44 and CD166 expression. Second, Twist1 induced activation of AKT and suppression of GSK-3β, which resulted in activation of β-catenin, thereby increasing CD44 expression. The AKT/NF-κB pathway and AKT/GSK-3β/β-catenin pathway activated in the MSS LS513 cells, while only the AKT/GSK-3β/β-catenin pathway activated in the MSI LoVo cells in response to Twist1 overexpression (Figure 7).

Twist1, the basic helix-loop-helix transcription factor, is a potent promoter of cancer progression and metastasis [21]. It is a key regulator of EMT and promotes cancer cells to display mesenchymal phenotypes such as a spindle-like shape, proliferation, migration, and invasion [6, 7, 21–23]. Several studies have suggested that Twist1 is associated with pathways in the EMT process such as Wnt/β-catenin signaling [24], PI3K/AKT/TGF-β signaling [25, 26], AKT2 signaling [20], and NF-κB signaling[21] in breast cancer cells. However, the mechanisms by which Twist1 promotes EMT are still poorly understood. Many studies have shown that cancer cells can have stem cell-like characteristics during EMT, which results in colony formation and expression of stem cell markers [27–30]. CSCs known as tumor-initiating cells have been identified in several tumors [31–33]. These cells have characteristics such as self-renewal, tumor formation, and resistance to therapy, so they lead to cancer recurrence and metastasis [30, 34, 35].

MSI results from defective DNA mismatch repair, which is one of the major mechanisms of carcinogenesis [16, 17]. MSS CRC patients experience more frequent metastasis and worse prognosis compared to MSI CRC patients [18, 19]. In a study of 2,141 CRC patients, distant metastases were more frequent in MSS patients than in MSI patients (22% *vs*. 12%) [36]. Many studies have identified these prognostic differences between MSS and MSI CRC patients, but little is known about the underlying mechanisms. Recent studies have suggested that EMT is impaired in colon cancer with MSI compared to that with MSS [4, 37, 38]. However, a signaling pathway according to MSI status was not identified.

We hypothesized that differences in EMT signaling pathways might be one of the mechanisms that cause prognostic differences according to the MSI status. In our study, Twist1 effectively induced the EMT process in MSS LS513 cells. In contrast, Twist1 induced only a partial EMT process in MSI LoVo cells, and the following phenomena occurred. First, the epithelial marker E-cadherin was downregulated, but the mesenchymal marker vimentin was not upregulated. Second, cell mobility and invasiveness were not effectively increased. Third, the AKT/GSK-3β/β-catenin pathway was activated, but not the AKT/NF-κB pathway. Finally, expression of CD44 increased, not CD166. These results provide experimental evidence for the establishment of therapeutic strategies targeting EMT pathways according to MSI status.

There were some limitations to our study. We used one cell line each with an MSS and MSI phenotype, respectively. In addition, we did not describe the reason why the AKT/NF-κB pathway did not activate in the MSI cell line. However, this study is the first focusing on the EMT signaling cascade according to MSI status. In addition, we confirmed *in vitro* results with additional xenograft experiments, suggesting the possibility of applying these results to human cancers. Cancer cells with stem cell-like characteristics are induced by EMT and have therapeutic resistance. These cells can repopulate primary tumors, resulting in cancer recurrence and metastasis. Thus, selective targeting of these cells is needed. Our results suggest that targeting the AKT/NF-κB and AKT/GSK-3β/β-catenin pathways can suppress the stem cell-like characteristics associated with Twist1-induced EMT. Furthermore, these pathways are different according to MSI status, so a different approach is needed for these two groups. Of note, inhibition of the EMT pathway effectively suppressed cell invasiveness in the MSS phenotype corresponding to the majority of CRC cases, which can help improve survival in CRC patients.

In conclusion, Twist1 induces stem cell-like characteristics by EMT via AKT signaling pathways in colon cancer cell lines, and these pathways depend on the MSI status. This study may be useful for developing new therapeutic strategies according to MSI status and may help to improve survival outcome in CRC patients.

## MATERIALS AND METHODS

### Cell cultures and viral transduction

MSS LS513 and MSI LoVo colon cancer cell lines were cultured in an RPMI 1640 medium (Gibco, Grand Island, NY, USA) supplemented with 10% fetal bovine serum (FBS; Gibco), and 1% penicillin-streptomycin (Gibco) in a 5% CO_2_ incubator at 37°C. The medium was replaced every 2 days. Lentiviruses expressing human Twist1 and GFP were provided by Dr. Seok-Hyung Kim (Department of Pathology, Samsung Medical Center) [39]. For lentiviral transduction of LS513 and LoVo cells, 100 multiplicity of infection Twist1 or GFP lentiviruses were added to a well containing 5×10^4^ cells, medium and 8 μg/ml polybrene. After 24 hours of incubation, transduced cells were selected with 1,000 μg/ml hygromycin (Sigma, St. Louis, MO, USA). GFP lentivirus was used as a control. Selected cells were maintained in growth medium with 500 μg/ml hygromycin in a 5% CO_2_ incubator at 37°C.

### Western blot

To prepare whole-cell extracts, cells were lysed using a protein lysis buffer (Pro-prep, Intron) including a protease inhibitor. Then, 40–60 μg of protein extract were incubated with primary antibodies against Twist1 (ab50887, Abcam), E-cadherin (catalog # 610181, BD), vimentin (sc-32322, Santa Cruz), total β-catenin (catalog # 610154, BD), phospho-β-catenin (CST-9561, Cell Signaling), total AKT (CST-4691, Cell Signaling), phospho-AKT (CST-4060, Cell Signaling), total phospho-GSK3 (CST-9315, Cell Signaling), phospho-GSK3 (04-1075, Millipore), IKKα (CST-2682, Cell Signaling), IKKβ (CST-2370, Cell Signaling), IκBα (sc-371,Santa Cruz), phospho-IκBα (CST-2859, Cell Signaling), p65 (sc-8008, Santa Cruz), and CD44 (CST-3570, Cell Signaling), followed by incubation with goat anti-mouse IgG or goat anti-rabbit IgG secondary antibodies conjugated to horseradish peroxidase (Santa-Cruz). Fractionation was performed using NE-PER Nuclear and Cytoplasmic Extraction Reagents (#78833, Thermo Fisher, San Jose, CA) via sequential extraction of cytosolic and nuclear proteins in non-ionic detergent for investigation of b-catenin and NF-kB localization. Cells were lysed with cytoplasmic lysis buffer for 15 min on ice then spun for 5 min to collect cytosolic lysate. Pellets were washed with cytoplasmic lysis buffer, and then lysed with nuclear lysis buffer for 40 min on ice. The lysates were spun for 10 min at 12,000xg at 4°C to collect nuclear lysates. β-actin (CST-3700, Cell Signaling) and laminB (Sc-6216, Santa Cruz) were used as cytosolic/total and nuclear normalized protein controls, respectively, in Western blotting.

### Quantitative real-time PCR

Total RNA was extracted from Twist1-transfected or untransfected cells (RNAprep Mini kit, Qiagen), and 500 ng RNA was subjected to reverse transcription using MuLV reverse transcriptase (NEB). Real-time quantitative PCR amplification was performed with a two-step TaqMan Probe Master Mix (Roche, Germany) in a real-time system (ABI, USA). Human-specific TaqMan PCR primers and probes (Roche, Germany) were used to analyze expression of the following genes: *Twist1*, *E-cadherin, β-catenin*, *vimentin*, *AKT*, *GSK-3β*, *IκBα*, *p65*, *CD44*, *CD166*, and *β-actin* (Supplement Table S1). mRNA levels of specific genes were calculated as ΔΔCt and normalized to β-actin.

### Cell migration and invasion assays

Cell migration assays were carried out using uncoated Matrigel transwell migration chambers (BD Bioscience, CA) in 24-well cell culture plates. In contrast, cell invasion assays were carried out using a Matrigel invasion chamber (BD Bioscience, CA) hydrated for at least 2 hour with 500 μl serum-free RPMI in the bottom of the well and 500 μl in the top of the chamber. After hydration of the Matrigel, DMEM in the bottom of the well was replaced with 700 μl of RPMI containing 10% FBS. Stable cells (5×10^4^/well) were loaded in migration and invasion chambers with 500 μl of serum-free RPMI medium. In the lower chambers, 700 μl of RPMI supplemented with 10% FBS was added as a chemo-attractant. Plates were incubated for 24 or 48 hours and then stained with calcein (2 μM, BD Biosciences, CA). Cell migration and invasion were quantified using fluorescence with a VICTOR2 Multilabel Counter (Perkin Elmer) equipped with a 485/520 nm filter set.

### Soft agar colony formation assay

A colony-forming mixture containing 1.2% agar solution, 2×DMEM medium without serum, and 1,500 μl of stable cell (2 × 10^4^/well) suspension (1:1:1) was added to 24-well plates, and plates were incubated for 8–10 days. Colonies were photographed with an inverted microscope (Olympus, USA), and cell growth was quantified based on the number and size of the colonies.

### FACS

Cells were suspended in phosphate buffered saline (PBS) and incubated with an FcR blocking reagent (Miltenyi Biotec, Germany) for 10 minutes. Then, cells were stained with the directly conjugated monoclonal antibodies anti-human CD166-PE, CD44-APC, anti-human IgG-PE isotype, and anti-human IgG-APC isotype (Miltenyi Biotec, Germany) for 30–40 minutes at 4 °C. The IgG isotype control was incubated in parallel. Flow cytometry was performed with an Accuri C6 (BD Biosciences, CA) using the CFlow software (BD Biosciences, CA).

### Immunofluorescent staining

Sterile cover slips were placed in 12- or 24-well plates and then rinsed with PBS, followed by a quick rinse with culture medium. Cells were plated on the cover slips at a density of approximately 10,000/cm^2^ and then fixed in a freshly prepared solution of 4% formaldehyde in PBS and quenched with 50 mM NH_4_Cl for 15 minutes. Cells were blocked and permeabilized (if the first antibody was against a cytoplasmic domain of the protein or was present intracellularly) for one hour at room temperature. Next, the cells were incubated with the directly conjugated monoclonal antibodies anti-human CD166-PE, CD44-APC, and anti-mouse IgG isotype in a blocking/permeabilization solution overnight at 4 °C. After incubation, the cover slips were rinsed with distilled water and incubated with the secondary antibody Alexa488 (Miltenyi Biotec, Germany) to rule out nonspecific binding and cross-reaction between the secondary antibodies. The negative control was incubated with mouse IgG instead of the first antibody, followed by incubation with a secondary antibody to check for nonspecific binding. Images of immunofluorescence were analyzed with a confocal LSM700 microscope (Zeiss, Germany).

### Antibody neutralization

Stable cells were pre-incubated in a serum-free medium containing an RPMI-supplemented neutralization antibody against CD44 or CD166 (1:10 dilution, Mitenyi Bitotec) for one hour. These cells (5×10^4^/well) were used in an *in vitro* invasion assay using BioCoat Matrigel invasion chambers (BD, Biosciences, CA). IgG was used as a control for antibody neutralization. After that, a cell invasion assay was performed by staining with calcein; fluorescence was quantified with a VICTOR2 Multilabel Counter (Perkin Elmer) equipped with a 485/520 nm filter [40].

### Xenograft experiments

We examined tumorigenesis *in vivo*, using an assay to evaluate cancer growth induced by Twist1 overexpression. Cells (5 × 10^6^/100μl) overexpressing Twist1 were suspended in 50 μl of PBS supplemented with 50% Matrigel and injected subcutaneously into the flanks of 6-week-old female BALB/c nu/nu mice (Charles River Laboratories, Wilmington, USA). Tumor size was measured once a week with a caliper, and tumor volume was calculated using the following formula: short length^2^ x long length/2. Mice were sacrificed 3–4 weeks after inoculation or as soon as a reduction in vitality was observed. Animal experiments were reviewed and approved by the Institutional Animal Care and Use Committee (IACUC) of the Samsung Biomedical Research Institute (SBRI) (#20150122002). SBRI is an Association for Assessment and Accreditation of Laboratory Animal Care International (AAALAC International)-accredited facility and abides by the Institute of Laboratory Animal Resources (ILAR) Guide.

### Statistical analysis

All data are presented as the mean ± standard deviation. Student's *t* tests comparing data from each experiment were performed using the SigmaPlot version 10 software (Systat Software Inc.). All experiments were done at least three times. *P*-values less than 0.05 were considered statistically significant.

## SUPPLEMENTARY MATERIALS TABLE


