# Supplementary material for: Twist1-induced epithelial-mesenchymal transition according to microsatellite instability status in colon cancer cells
Source: Oncotarget. 2016 Aug 1;7(35):57066–76. doi: 10.18632/oncotarget.10974 (PMC5302973; doi:10.18632/oncotarget.10974)
Supplement: Supplementary file 1 [file oncotarget-07-57066-s001.pdf]

Twist1-induced epithelial-mesenchymal transition according to microsatellite instability status in colon cancer cells

SUPPLEMENTARY TABLE

Supplementary Table S1: Primer sequences and conditions used in real-time PCR reactions

Target gene	Primer sequence (Taqman probe)	Number of cycles	Annealing temperature (°C)	Expected fragment size (bp)
β-actin	CCTTTGCCGATCCGCCGCCCGTCCA	40	60	85
Twist1	CGGAGACCTAGATGTCATTGTTTCC	40	60	85
RELA(p65)	AGTACCTGCCAGATACAGACGATCG	40	60	85
IKKα	TATGATGAATCTTGATTGGAGTTGG	40	60	85
IKKβ	CCGTGCAGTGGCATTCAAAAGTGCG	40	60	85
IκBα	GACGGGGACTCGTTCCTGCACTTGG	40	60	85
E-cadherin	CGCGTCCTGGGCAGAGTGAATTTTG	40	60	85
Vimentin	CTCCGGGAGAAATTGCAGGAGGAGA	40	60	85
β-catenin	CGCCAGGATGATCCTAGCTATCGTT	40	60	85
AKT	CTCCTGAGGAGCGGGAGGAGTGGAC	40	60	85
GSK3β	AGCATGAAAGTTAGCAGAGACAAGG	40	60	85
CD44	TCGAAGAAGGTGTGGGCAGAAGAAA	40	60	85
CD166	AATGGAAATATGAAAAGCCCGATGG	40	60	85
